# Disparities in spinal deformity surgery care for children with cerebral palsy and neuromuscular scoliosis

**DOI:** 10.1007/s43390-025-01198-6

**Published:** 2025-10-17

**Authors:** Luis Torres-Gonzalez, Sara J. Morgan, Christopher D. Seaver, Rhonda G. Cady, Zelphia C. Brown, Maykala J. Williams, Daniel J. Miller

**Affiliations:** 1 Research Department, Gillette Children’s Specialty Healthcare, 200 University Avenue East, St. Paul, MN 55101 USA; 2 Department of Family Medicine and Community Health, University of Minnesota, Minneapolis, MN USA; 3 School of Nursing, University of Minnesota, Minneapolis, MN USA; 4 Department of Orthopaedics, Gillette Children’s Specialty Healthcare, St Paul, MN USA; 5 Department of Orthopaedic Surgery, University of Minnesota, Minneapolis, MN USA

**Keywords:** Neuromuscular scoliosis, Health disparities, Pediatrics, Spine surgery

## Abstract

**Purpose:**

To assess potential disparities in care for non-ambulatory children with cerebral palsy (CP) and associated neuromuscular scoliosis treated at a quaternary pediatric hospital.

**Methods:**

This retrospective cohort study included non-ambulatory children CP who received spinal deformity surgery between 01/2012 and 12/2022. Demographic, clinical, and radiographic data were collected. Relationships between demographic factors and clinical/radiographic data were assessed using Fisher’s Exact Test, Wilcoxon Rank-Sum test, Kruskal–Wallis one-way ANOVA, and linear regression models.

**Results:**

Of 502 children identified, 328 met eligibility criteria. The mean age of the sample was 9.8 ± 4.0 years, 59% were male. On presentation, the average major coronal curve magnitude was 46 ± 23˚. Most (70%) were White, 13% were Black, 6% were Hispanic or Latino, and the remaining participants were Asian, Pacific Islander, Native American, Alaska Native, or declined to answer. Most spoke English (89%). Just under half (45%) had both government and commercial insurance and 24% had only government insurance. Black compared to White race (*p* = .03), government compared to commercial insurance (*p* = .02), and farther distance from hospital (*p* < .001) were associated with larger curve magnitudes at presentation, after adjustment for covariates. Non-English language (*p* = .002) was associated with longer time from surgical recommendation to surgery, after adjustment for covariates.

**Conclusions:**

Health disparities were identified related to ethnicity, race, preferred language, and geographical distance from the hospital for children with CP and neuromuscular scoliosis. These findings highlight the need for development of standardized criteria for surveillance, imaging, and referral to reduce health disparities for this specific population.

## Introduction

Non-ambulatory children with neuromuscular conditions, such as cerebral palsy (CP), often have severe progressive scoliosis. Neuromuscular scoliosis is found in 15–80% of children with CP whereas idiopathic scoliosis occurs in only 1–2% of the general population [1]. Curve progression past maturity is more common in children with neuromuscular disease compared to idiopathic scoliosis, and children with neuromuscular scoliosis are also more likely to have increased pelvic incidence, which can cause discomfort with seating and posture [2]. In non-ambulatory children, non-operative treatments rarely prevent progression of the spinal curve. As such, spinal deformity surgery is often used to treat severe and progressive spinal curvatures [1, 2].

Sociodemographic factors have historically impacted healthcare in the USA. In pediatric orthopedics, disparities in access to care and treatment options have been demonstrated based on sociodemographic factors such as race, socioeconomic status (SES), and type of health insurance for children with a variety of conditions [3–6]. For example, two studies conducted in different regions of the USA found that Black children with adolescent idiopathic scoliosis (AIS) were more likely to have a surgical magnitude curve at presentation and have surgery as their initial treatment [4, 6], and this effect was even stronger for Black children on government insurance [4]. Another study found that children with AIS who lived in rural areas had higher rates of readmission and instrumentation removal compared to those who lived in urban areas [7]. For children with CP and hip dysplasia, Black children were significantly more likely to undergo a hip salvage procedure for hip dysplasia versus a reconstructive hip procedure when compared to White children, and Black race was also found to be an independent risk factor for medical complications [3]. Further, children with government-funded insurance experienced significant delays in access to care for fractures compared to those on commercial insurances [8].

Given these prior findings in pediatric orthopedics, and notable health disparities for children with developmental disabilities [9–12], it is hypothesized that similar disparities are present for children with neuromuscular scoliosis. However, to our knowledge, no prior studies have investigated the relationship between demographic characteristics and treatment indicators in this population. Thus, this study aims to identify potential disparities in access to care for children with neuromuscular scoliosis associated with non-ambulatory CP who were treated at a single quaternary pediatric hospital. Results from this study will inform strategies to increase equity in care for these children.

## Methods

A retrospective cohort study of children with a diagnosis of neuromuscular scoliosis and non-ambulatory CP was conducted at a single quaternary hospital in Minnesota, located in the midwestern United States. This study was approved by an institutional review board.

### Participants

Participants were identified using diagnostic codes for CP and scoliosis and procedural codes for spinal deformity surgery. The list was narrowed by removing those that opted out of medical chart review. The remaining children were assessed for eligibility through retrospective review of the medical records. Eligibility criteria were: (1) diagnosis of CP and neuromuscular scoliosis, (2) non-ambulatory as designated as Gross Motor Function Classification System (GMFCS) of IV or V, (3) spinal deformity surgery between January 2012 and December 2022 at a single institution, and (4) under the age of 21 years at the time of index surgery. Children were also excluded if they received prior spine deformity-related treatment prior to presentation to this center.

### Data abstraction

Demographic and clinical data were collected from electronic medical records. Demographic data included ADI, self-reported race/ethnicity, self-reported preferred language, health insurance, and distance to the hospital. Clinical data included curve magnitude at presentation, corrective surgery type, and time from surgery recommendation to surgery. The variable of race/ethnicity was collapsed into the categories of White, Black or African American, Hispanic or Latino, and other (i.e., Asian, Alaskan Native or American Indian, or other) for analyses. The other race/ethnicity category was created due to small cell sizes for those racial/ethnic categories. Individuals who declined to provide their race/ethnicity were excluded from the analyses. Health insurance types include the categories of government, commercial, commercial/government combination, or self-pay. The Area Deprivation Index (ADI) is a metric that measures whether a neighborhood is socioeconomically disadvantaged [13]. National ADI values for each child were obtained from the University of Wisconsin School of Medicine and Public Health’s Neighborhood Atlas [13], an online tool designed to provide a value for socioeconomically disadvantaged neighborhoods based on several factors of social health, including educational level, income, and housing quality. Higher percentiles indicated more disadvantaged neighborhoods. Distance from hospital was the shortest distance between the child’s home and the hospital location on Google Maps (Google, Mountain View, CA), in miles.

Curve magnitude was measured as the major curve angle on seated coronal radiographs at the time the child first presented at our institution for specialty spine care. Radiographic measurements were performed by research personnel trained by pediatric orthopedic spine surgeons. Time from recommendation to surgery was calculated in months and served as a measure of time from surgical recommendation to the actual time of their corrective surgical procedure. Type of corrective surgery included the categories of spinal fusion and growth-friendly surgery (traditional or magnetically controlled growing rods).

All data were recorded and managed in a Research Electronic Data Capture (REDCap) database (Vanderbilt University, Nashville, TN) [14, 15]. Data accuracy was assessed by two investigators and training was provided for each team member involved in data collection.

### Statistical analysis

Descriptive statistics were calculated for all variables. Individual factor analyses to explore relationships between demographic factors and clinical/radiographic data were assessed using Fisher’s Exact Test, Wilcoxon Rank-Sum test, Kruskal–Wallis one-way ANOVA and post-hoc Dunn’s Test, and simple linear regression models. Two multiple linear regression models were run to explore the relationship between a variety of demographic factors (while controlling for others) and clinical/radiographic data. A multiple binomial logistic regression model to explore the relationship between a variety of demographic factors and corrective surgery type was not included in the analysis, as cell sizes were too small to meet model assumptions and yield valid estimates. We confirmed the models met regression assumptions and evaluated their fit using AIC and adjusted R-squared values. This was a complete case analysis, for whom individuals with missing data were excluded from the analysis. Post-hoc analyses using Chi-square tests were conducted for statistically significant findings to determine the proportion of children who had preoperative curves over 90˚, a threshold related to clinical implications such as increased risk of infection, blood loss, and the need for anterior/posterior procedures [16]. For all analyses, the significance threshold was defined as a two-sided *P*-value < 0.05. The analysis was conducted using R Studio version 4.1.1 (2021.08.10) [34].

## Results

### Participant characteristics

**Fig. 1:**
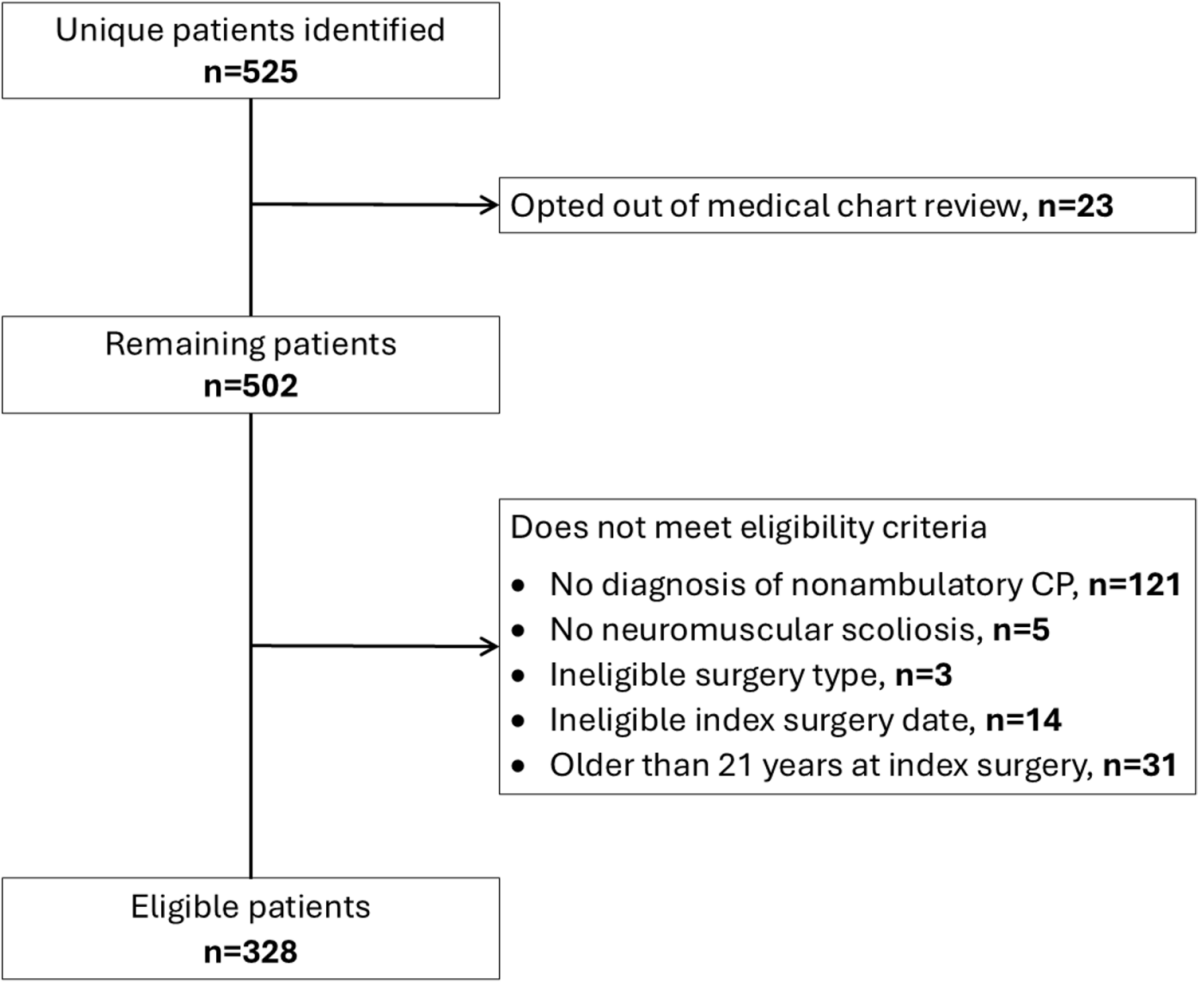
Flow chart for children eligible for the study

A total of 328 participants were eligible for this study (Fig. 1). Descriptive statistics of study participants are presented in Tables 1 and 2.

**Table 1:** Sample characteristics (*N* = 328)

Characteristic	*N*	%
Sex		
Female	133	40.5
Male	195	59.9
Race/ethnicity		
Asian/Pacific Islander	10	3.0
Black	44	13.4
Hispanic/Latino	20	6.1
American Indian/Alaska Native	4	1.2
White	230	70.1
Other	10	3.0
Information not available	10	3.0
Preferred language		
English	293	89.3
Non-English	35	10.7
Insurance		
Commercial	61	18.6
Commercial/Government	146	44.5
Government	80	24.4
Self	38	11.6
Type of corrective surgery		
Fusion	284	86.6
Growth-friendly	44	13.4
	Mean	SD
Age, years	9.8	4.0
Area Deprivation Index (ADI)	45.0	20.2
Distance, miles	104.6	149.3
Curve magnitude at presentation, degrees	46	23
Length of time to surgery, months	8.4	7.3

**Table 2:** Descriptive statistics of study participants by race/ethnicity, preferred language, and insurance type

	Race/ethnicity				Language		Insurance type			
	White *N* = 230)	Black/A *(N* = 44)	Hispanic/Latin *(N* = 20)	Other *(N* = 24)	English *(N* = 293)	Non-English *(N* = 35)	Comm *(N* = 61)	Comm/Gov *(N* = 146)	Gov *(N* = 80)	Self *(N* = 38)
Curve magnitude at presentation, degrees										
Mean (SD)	44 (22)	52 (30)	50 (26)	50 (24)	45 (23)	51 (29)	40 (21)	47 (23)	49 (22)	43 (27)
Med (IQR)	41 (29)	46 (41)	38 (36)	48 (33)	41 (31)	46 (28)	34 (25)	42 (32)	48 (28)	36 (30)
Length of time to surgery, months										
Mean (SD)	8.0 (6.9)	10.3 (8.3)	8.2 (8.5)	9.3 (9.0)	7.9 (6.9)	12.3 (9.5)	7.3 (4.0)	8.4 (7.8)	8.3 (7.0)	9.5 (9.2)
Med (IQR)	6.0 (5.4)	7.2 (9.9)	5.0 (3.7)	6.2 (6.3)	6.0 (5.5)	8.3 (12.7)	6.1 (4.8)	6.3 (5.3)	5.5 (6.4)	5.9 (8.8)
Distance, miles										
Mean (SD)	123.1 (144.0)	78.4 (216.3)	45.1 (64.6)	54.5 (80.1)	114.6 (154.8)	20.4 (20.3)	87.9 (106.6)	125.8 (185.6)	87.7 (116.1)	81.9 (103.3)
Med (IQR)	74.7 (130.3)	19 (32.0)	19.6 (26.9)	20.9 (46)	58.5 (117.1)	15.2 (14.2)	41.4 (85.3)	61.7 (132.1)	39.8 (89.5)	26.7 (78.4)
ADI										
Mean (SD)	44.5 (20.4)	48.5 (17.9)	50.5 (21.9)	45.7 (21.1)	44.7 (20.5)	47.0 (17.2)	38.8 (18.2)	46.2 (19.4)	45.3 (20.6)	48.0 (22.6)
Med (IQR)	43 (27)	47 (19.8)	49 (25)	42 (23.8)	43 (27)	44 (18.5)	39 (29)	44 (24)	44 (23.3)	43 (32)
Corrective surgery										
	–	–	–	–	-	–	–	–	–	–
Fusion										
* N* (%)	195 (84.8)	43 (97.7)	17 (85.0)	20 (83.3)	250 (85.3)	34 (97.1)	53 (86.9)	121 (82.9)	72 (90.0)	35 (92.1)
Growth-friendly										
* N* (%)	35 (15.2)	1 (2.3)	3 (15.0)	4 (16.7)	43 (14.7)	1 (2.9)	8 (13.1)	25 (17.1)	8 (10.0)	3 (7.9)

Values represent Mean (SD) and Median (IQR) for continuous variables, *N* (%) for categorical variables

*AA* African American, *ADI* Area Deprivation Index, *comm* Commercial, *gov* Government; *med* Median

### Relationships between sociodemographic and clinical factors

#### Curve magnitude at presentation

**Fig. 2:**
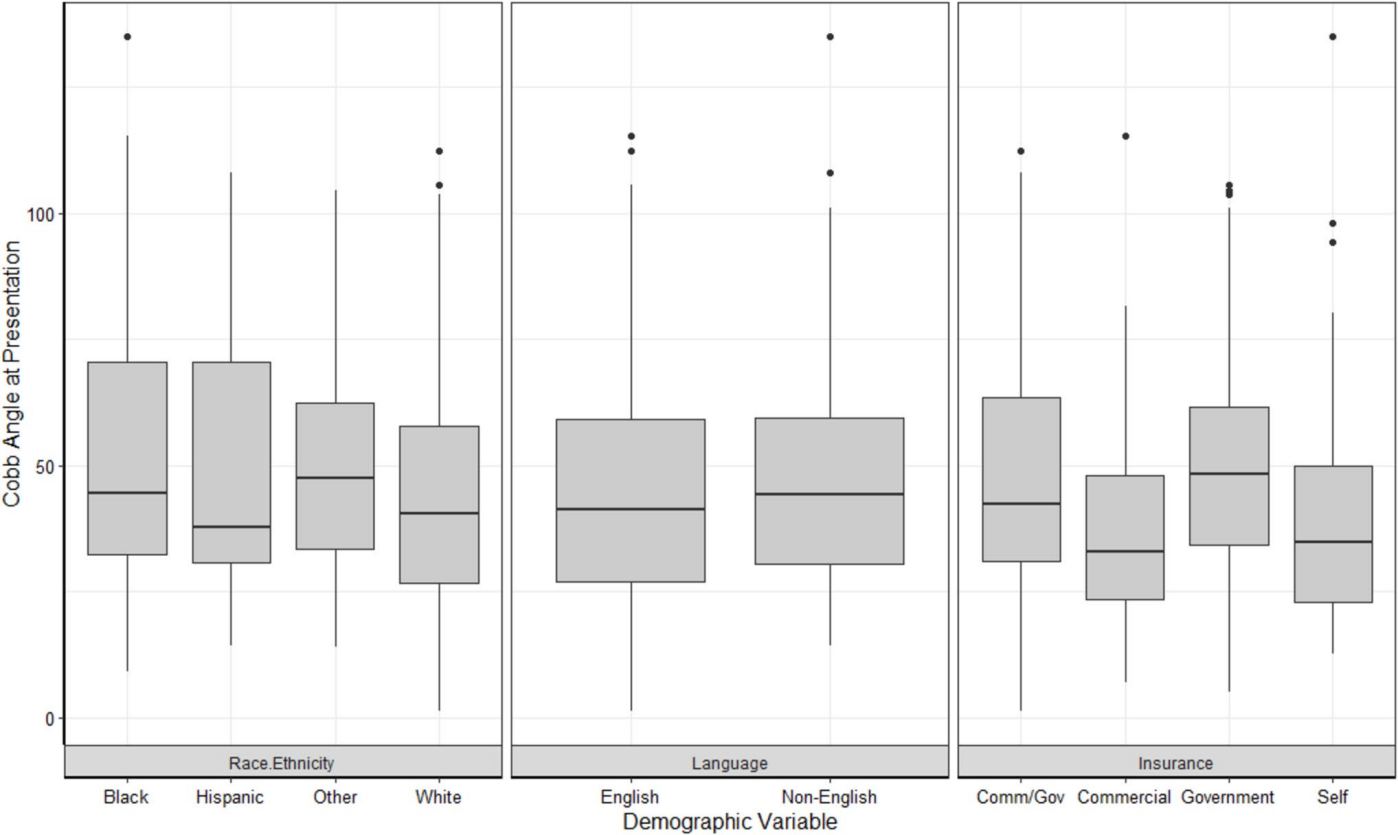
Curve magnitude at presentation by race/ethnicity, preferred language, and insurance type

In individual factor analyses, significant relationships were present between curve magnitude at presentation and both health insurance at the first spine visit (*p* = 0.02) and distance from treating hospital (*p* < 0.001). Post-hoc analyses demonstrated significant differences in curve magnitude at presentation between participants with commercial insurance and those with government insurance (adjusted *p* = 0.04). When the demographic variables were entered into a multiple linear regression model, the model was significant for race (Black or African American different than White, *p* = 0.03), health insurance (commercial different than government, *p* = 0.02), and distance from treating hospital (*p* < 0.001). Black children had, on average, a curve that was 9˚ larger at presentation than White children and children with government insurance had, on average, a curve that was 9˚ larger at presentation than children with commercial insurance when controlling for other variables in the model. With respect to distance, for every additional mile away from the hospital, there was an increase in curve magnitude by 0.03˚ (equating to increases of 3˚ for every 100 miles) when controlling for other variables in the model (Table 3, Fig. 2).

**Table 3:** Multiple linear regression results of the relationship between socio-demographic factors and clinical factors (i.e., curve magnitude at presentation and length of time (months) from surgical recommendation to surgery)

	Curve magnitude at presentation (degrees)		Length of time to surgery (months)	
	Estimate (SE)	*p*-value	Estimate (SE)	*p*-value
Race				
White	Ref	Ref	Ref	Ref
Black or AA	8.87 (4.13)	0.03*	0.61 (1.30)	0.64
Hispanic or Latino	6.92 (5.85)	0.24	− 2.61 (1.85)	0.16
Other	9.27 (5.14)	0.07	− 0.11 (1.62)	0.95
Primary language				
English	Ref	Ref	Ref	Ref
Non-English	2.58 (4.95)	0.60	4.85 (1.56)	0.002*
Insurance				
Commercial	Ref	Ref	Ref	Ref
Commercial and Government	6.67 (3.53)	0.06	1.66 (1.11)	0.14
Government	8.95 (3.96)	0.02*	0.70 (1.25)	0.57
Self-pay	4.08 (4.78)	0.40	2.14 (1.51)	0.16
Distance	0.03 (0.01)	< .001*	− 0.004 (0.003)	0.19
Area Deprivation Index	0.0001 (0.07)	1.00	0.01 (0.02)	0.72

^*^denotes statistical significance at *p* < .05

#### Time from surgical recommendation to surgery

**Fig. 3:**
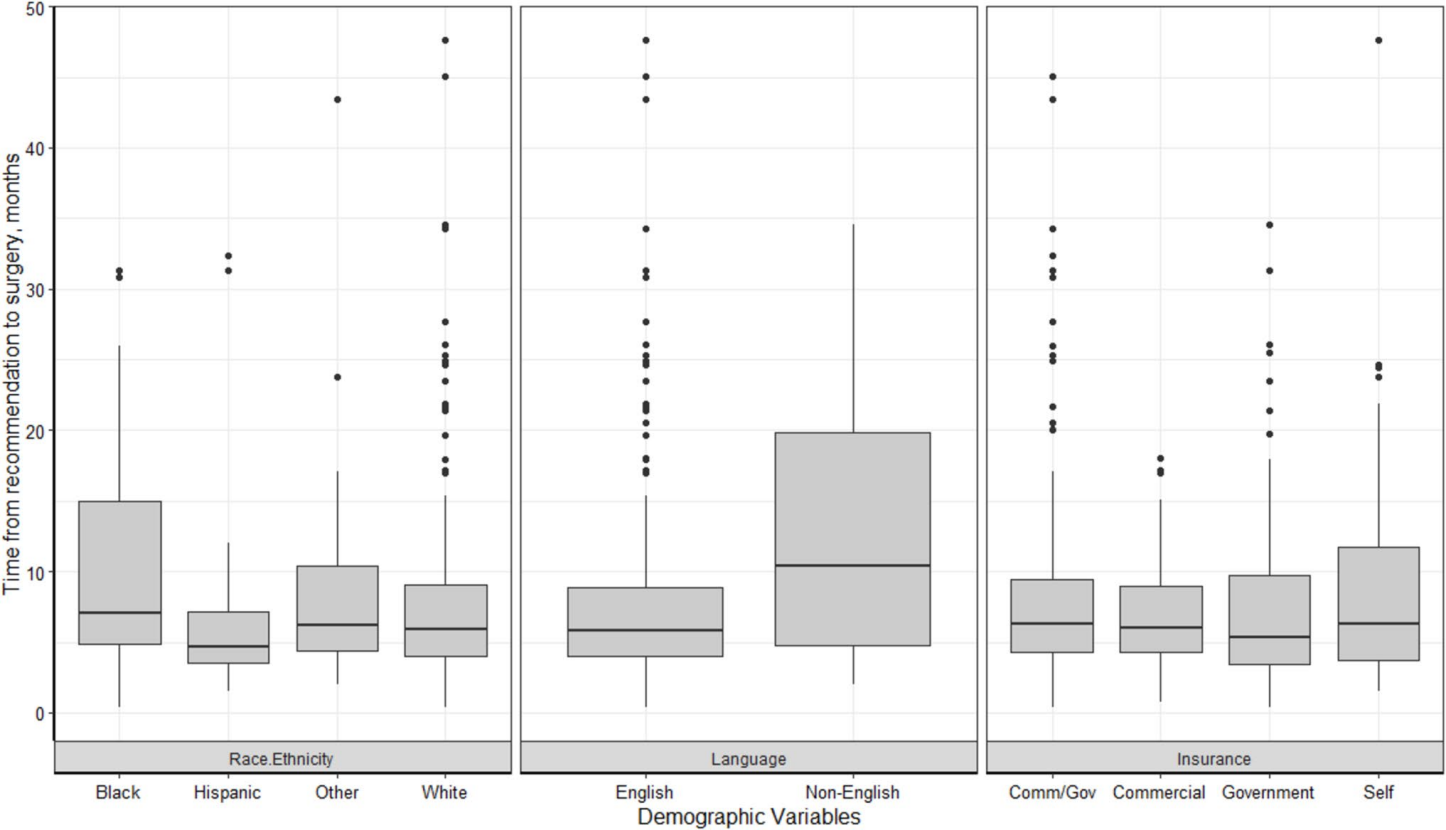
Time from surgical recommendation to surgery by race/ethnicity, preferred language, and insurance type

In individual factor analyses, significant relationships were present between time from surgical recommendation to surgery for preferred language (*p* = 0.005) only, with non-English speakers having a longer average time from surgical recommendation to surgery. When the demographic variables were entered into a multiple linear regression model, the model was still significant for language (*p* = 0.002), with an average of 4.9 months longer between surgical recommendation and surgery for non-English speakers than English speakers when controlling for other variables in the model (Table 3, Fig. 3).

#### Preoperative curve magnitude over 90˚

There were significant differences in the proportion of children who had severe preoperative curves (over 90˚) for Black children compared to White children (41% vs. 23%, *p* = 0.01) and children who did not speak English as their preferred language compared to children who did (44% vs. 25%, *p* = 0.02). Non-significant differences in proportions were observed for children on government insurance compared to commercial insurance (28% vs. 16%, *p* = 0.11) and children who lived > 200 miles from the treating hospital (32% vs. 26%, *p* = 0.38).

#### Type of corrective surgery

We were unable to conduct analyses due to small cell sizes, and thus resulting estimates may not be accurate. However, it is noteworthy that a smaller percentage of Black (2%) and non-English-speaking children (3%) received growth-friendly surgery compared to other races (15–17%) and English-speaking children (15%) (Table 2).

## Discussion

This study assessed relationships between demographic characteristics and indicators of healthcare availability for children with non-ambulatory CP and neuromuscular scoliosis. To our knowledge, this is the first study to investigate health disparities related to spinal deformity care in this population. Our findings indicate significant relationships between sociodemographic factors and indicators of healthcare availability.

Larger major coronal curves at presentation were significantly related to Black race, government health insurance type, and longer distance between the child’s house and the hospital. While there are no prior studies that assess these relationships in children with neuromuscular scoliosis, our findings are similar to studies of children with AIS, which found that Black children with AIS presented with larger curves at the initial visit [4, 6]. Recent studies reported conflicting findings relating to type of health insurance and curve magnitude at presentation in children with AIS, but several have found that children with public (government) insurances presented with surgical-level curves [4, 17–19], similar to our findings of larger curve magnitudes for children with CP on government health insurance. In addition, longer distances from a person’s home to hospitals have been associated with worse outcomes in people who have sustained strokes, colorectal cancer, and life-threatening emergencies [20–22]. However, only one study [23] assessed how distance from the treating hospital is related to curve severity in children with AIS. Unlike our study which found that children with CP had larger curves at presentation, there was no significant association between distance and curve severity in children with AIS. This difference in findings between these two patient groups may be because most children with AIS have no other health conditions and the family can prioritize the care needed for AIS [23]. Larger major coronal curves at presentation indicate that children with CP and their families face obstacles accessing the specialty healthcare that they need. Data from the present study cannot be used to explore underlying mechanisms or barriers faced by children with neuromuscular scoliosis and their families. Future qualitative and prospective studies may be beneficial to strengthen our understanding of the patient and family experience with barriers to care and provide context for the disparities in care identified in this study.

Limited studies have investigated the relationship between curve magnitude at presentation and preferred language for patients with scoliosis, although one indicated that preferred language had no relationship with curve severity at presentation for children with AIS [24]. Historically, however, language barriers have been associated with poorer outcomes not only in orthopedics but also multiple aspects of healthcare [25, 26]. Language and cultural barriers can result in medical mistrust and delays due to apprehension about surgical treatments. In the current study, the time between recommendation and surgery was approximately five months longer on average and a larger percentage of patients with curves over 90˚ for children with a non-English preferred language compared to those who reported English as their preferred language. Delays in surgery have been shown to result in clinically-significant curve progression in skeletally immature children with AIS [27]. The delay observed may be multifactorial, as the process of preparing for a spinal fusion or growth-friendly surgery is extensive, expensive, and complex resulting in a higher potential for coordination of services and cancelations. It is important for individual healthcare systems to understand and address longer wait times for children and families who do not speak English as a primary language to proactively facilitate timely care.

We were unable to assess relationships between demographic characteristics and surgery type (e.g., fusion or growth-friendly surgery) because some cell sizes were too small and may result in inaccurate estimates. However, study findings suggest that smaller proportions of Black children and children who did not speak English as a primary language received growth-friendly rods (meaning they were more likely to receive fusion) compared to children in other racial groups. In the USA, structural and systemic inequities can present barriers to accessing necessary healthcare for Black Americans, as well as patients and families who rely on government health insurance or live in rural communities [28–32]. In current literature, racial disparities have been associated with limited access to care as well as poor surgical outcomes, notably for Black people. For example, Black people were found to have less access to common surgical treatments such as coronary bypass surgery, laparoscopic surgery, and breast reconstruction [33–35]. In orthopedics, clear differences have been identified in care for acute orthopedic trauma and postoperative outcomes for Black people compared to other racial groups [36, 37].

Nearly twice as many Black children and children with a non-English preferred language had curve magnitudes greater than 90˚ at the time of surgery compared to White children and children with English as their preferred language, demonstrating the clinical impact of delayed access to care or surgery. Preoperative curves over 90˚ are considered severe and are associated with increased risk of infection, blood loss, and the need for anterior/posterior procedures [16]. Scoliosis surveillance, imaging, and referral criteria for children with CP, similar to hip surveillance protocols for children with CP, [38] could reduce variation in treatment and minimize the potential influence of demographic factors (e.g., race and health insurance type). Scoliosis protocols will likely be based on factors such as the patients’ age, GMFCS level, and history of curve progression. To increase the likelihood of widespread adoption, such protocols need to be developed by multicenter and multidisciplinary teams in collaboration with national professional societies.

This study included patients from a single specialty healthcare center in a large metropolitan area (population 3.7 million). Our institution routinely provides scoliosis care to patients from a large catchment area that extends over 400 miles from our institution and includes urban, suburban, and rural patients from multiple surrounding states. While most of the patients in this sample were white (70%), our patient population is diverse with patients from multiple racial and ethnic groups, indigenous patients, and patients who are immigrants or refugees. The underlying reasons for demographic differences in access to timely healthcare in our region are not known but may be due to challenges with coordinating translation services or the cost and logistical considerations regarding traveling far distances for appointments. Findings may differ in regions where a greater percentage of the population is bilingual or institutions with a smaller catchment area. Similar studies in regions that differ from ours, as well as qualitative studies in our region will elucidate the underlying factors that contribute to the differences in care observed in this study.

### Limitations

This study has several limitations. Data collection occurred at a single specialty healthcare center in Minnesota. Children who seek care at our institution are largely White and speak English as their preferred language, and these demographic characteristics are similar to those in Minnesota. Thus, our findings cannot be generalized to other U.S. states with different demographic distributions and health systems. Studies in various regions around the country with more diverse populations will further elucidate the extent to which health disparities differ or present similarly at other institutions around the nation. Another limitation of the retrospective study design is the study team’s dependence on retrieving data from clinic notes where data may not be comprehensively recorded, which can impact both the quantity and quality of the data. However, the data we abstracted from medical records (e.g., appointment dates, demographic, and radiographic data) were generally robust and less prone to missingness. Finally, this study was only able to include children with neuromuscular scoliosis who received specialty spine care. Thus, the extent to which health disparities prevent children with neuromuscular scoliosis from receiving any specialty spine care is not known.

## Conclusion

These findings highlight potential disparities in healthcare access for non-ambulatory children with CP and neuromuscular scoliosis. Health disparities identified in this study included larger curve magnitudes at presentation for children who are Black, are on government insurance, and who live farther from the hospital. Other health disparities included a longer time between surgery recommendation to surgery for children who do not speak English as their preferred language. While additional studies are needed to better understand the nature of health disparities for children with neuromuscular scoliosis in other regions, these initial findings suggest the need for development of standardized criteria for surveillance, imaging, and referral to reduce health disparities for this specific population.

## Data Availability

The data that support the findings of this study are available upon reasonable request from the corresponding author.

## References

[1] Koop SE (2009) Scoliosis in cerebral palsy. Dev Med Child Neurol 51:92–98 19740215 10.1111/j.1469-8749.2009.03461.x

[2] Murphy RF, Mooney JF (2019) Current concepts in neuromuscular scoliosis. Curr Rev Musculoskelet Med 12:220–227 30941730 10.1007/s12178-019-09552-8 PMC6542926

[3] Brown L, Cho KM, Tarawneh OH, Quan T, Malyavko A, Tabaie SA (2022) Race is associated with risk of salvage procedures and postoperative complications after hip procedures in children with cerebral palsy. J Pediatr Orthop 42(9):e925–e931 35930795 10.1097/BPO.0000000000002216

[4] Heffernan MJ, Younis M, Song B, Fontenot B, Dewitz R, Brooks JT et al (2022) Disparities in pediatric scoliosis: the impact of race and insurance type on access to nonoperative treatment for adolescent idiopathic scoliosis. J Pediatr Orthop 42(8):427–431 35856501 10.1097/BPO.0000000000002213

[5] Mohanty S, Harowitz J, Lad MK, Rouhi AD, Casper D, Saifi C (2022) Racial and social determinants of health disparities in spine surgery affect preoperative morbidity and postoperative patient reported outcomes: retrospective observational study. Spine 47(11):781–791 35170553 10.1097/BRS.0000000000004344

[6] Zavatsky JM, Peters AJ, Nahvi FA, Bharucha NJ, Trobisch PD, Kean KE et al (2015) Disease severity and treatment in adolescent idiopathic scoliosis: the impact of race and economic status. Spine J 15(5):939–943 24099683 10.1016/j.spinee.2013.06.043

[7] Garcia SM, Niknam K, Sumandea F, Swarup I (2024) Socioeconomic differences in access to scoliosis care in the pediatric population. Spine Deform. 10.1007/s43390-024-00912-0 38898210 10.1007/s43390-024-00912-0

[8] Kitchen BT, Ornell SS, Shah KN, Pipkin W, Tips NL, Hogue GD (2020) Inequalities in pediatric fracture care timeline based on insurance type. JAAOS: Globa Res Rev 4(8):e20.00111 10.5435/JAAOSGlobal-D-20-00111 PMC7417144 32852914

[9] Cheak-Zamora NC, Thullen M (2017) Disparities in quality and access to care for children with developmental disabilities and multiple health conditions. Matern Child Health J 21(1):36–44 27423238 10.1007/s10995-016-2091-0

[10] Magaña S, Parish SL, Rose RA, Timberlake M, Swaine JG (2012) Racial and ethnic disparities in quality of health care among children with autism and other developmental disabilities. Intellect Dev Disabil 50(4):287–299 22861130 10.1352/1934-9556-50.4.287

[11] Colquitt G, Keko M, Rochani HD, Modlesky CM, Vova J, Maitre NL (2024) A cross-sectional study of disparities in healthcare transition in cerebral palsy. J Clin Med. 10.3390/jcm13133759 38999322 10.3390/jcm13133759 PMC11242745

[12] Weitzman C, Nadler C, Blum NJ, Augustyn M (2024) Health care for youth with neurodevelopmental disabilities: a consensus statement. Pediatrics. 10.1542/peds.2023-063809 38596852 10.1542/peds.2023-063809

[13] University of Wisconsin School of Medicine Public Health. Area Deprivation Index v2.4 2018 [Available from: http://www.neighborhoodatlas.medicine.wisc.edu. Accessed 22 Jan 2025

[14] Harris PA, Taylor R, Thielke R, Payne J, Gonzalez N, Conde JG (2009) Research electronic data capture (REDCap)—a metadata-driven methodology and workflow process for providing translational research informatics support. J Biomed Inform 42(2):377–381 18929686 10.1016/j.jbi.2008.08.010 PMC2700030

[15] Harris PA, Taylor R, Minor BL, Elliott V, Fernandez M, O’Neal L et al (2019) The REDCap consortium: building an international community of software platform partners. J Biomed Inform 95:103208 31078660 10.1016/j.jbi.2019.103208 PMC7254481

[16] Hollenbeck SM, Yaszay B, Sponseller PD, Bartley CE, Shah SA, Asghar J et al (2019) The pros and cons of operating early versus late in the progression of cerebral palsy scoliosis. Spine Deform 7(3):489–493 31053320 10.1016/j.jspd.2018.09.002

[17] Cho SK, Egorova NN (2015) The association between insurance status and complications, length of stay, and costs for pediatric idiopathic scoliosis. Spine 40(4):247–256 25494309 10.1097/BRS.0000000000000729

[18] Fletcher ND, Lazarus DE, Desai MJ, Patel NN, Bruce RW Jr (2015) Medicaid insurance is associated with larger curves in patients who require scoliosis surgery. Surgery 57(606):15–7 26566561

[19] Goldstein RY, Joiner ER, Skaggs DL (2015) Insurance status does not predict curve magnitude in adolescent idiopathic scoliosis at first presentation to an orthopaedic surgeon. J Pediatr Orthop 35(1):39–42 24978118 10.1097/BPO.0000000000000184

[20] Ader J, Wu J, Fonarow GC, Smith EE, Shah S, Xian Y et al (2019) Hospital distance, socioeconomic status, and timely treatment of ischemic stroke. Neurology 93(8):e747–e757 31320472 10.1212/WNL.0000000000007963 PMC6711658

[21] Thomas AA, Gallagher P, O’Céilleachair A, Pearce A, Sharp L, Molcho M (2015) Distance from treating hospital and colorectal cancer survivors’ quality of life: a gendered analysis. Support Care Cancer 23(3):741–751 25179691 10.1007/s00520-014-2407-9

[22] Nicholl J, West J, Goodacre S, Turner J (2007) The relationship between distance to hospital and patient mortality in emergencies: an observational study. J Emerg Med 24(9):665–668 10.1136/emj.2007.047654 PMC2464671 17711952

[23] Gilbert SR, Savage AJ, Whitesell R, Conklin MJ, Fineberg NS (2015) BMI and magnitude of scoliosis at presentation to a specialty clinic. Pediatrics 135(6):e1417–e1424 25963009 10.1542/peds.2014-2000

[24] Erkkila IP, Reynolds CA, Weissman JP, Levine OP, Aronson H, Knoll JM et al (2023) Factors associated with presentation of severe adolescent idiopathic scoliosis. J Pediatr Orthop Soc North Am 5(3):651 10.55275/JPOSNA-2023-651 PMC12088244 40433348

[25] Alley MC, Mason AS, Tybor DJ, Pevear ME, Baratz MD, Smith EL (2016) Ethnic barriers to utilization of total joint arthroplasty among Chinese immigrants in the United States. J Arthroplasty 31(9):1873–7 27026646 10.1016/j.arth.2016.02.046

[26] Flores G (2020) Language barriers and hospitalized children: are we overlooking the most important risk factor for adverse events? JAMA Pediatr 174(12):e203238-e 33074289 10.1001/jamapediatrics.2020.3238

[27] Ramo B, Tran D-P, Reddy A, Brown K, Niswander C, Erickson M et al (2019) Delay to surgery greater than 6 months leads to substantial deformity progression and increased intervention in immature adolescent idiopathic scoliosis (AIS) patients: a retrospective cohort study. Spine Deform 7(3):428–435 31053313 10.1016/j.jspd.2018.09.012

[28] Garg A, Lobner K, Song J, Mitchell R, Egbunine A, Kudchadkar SR (2023) Social determinants of health in pediatric rehabilitation for children with traumatic injury: a systematic review. J Pediatr 259:113459 37172806 10.1016/j.jpeds.2023.113459 PMC10524504

[29] Lechtholz-Zey E, Bonney PA, Cardinal T, Mendoza J, Strickland BA, Pangal DJ et al (2022) Systematic review of racial, socioeconomic, and insurance status disparities in the treatment of pediatric neurosurgical diseases in the United States. World Neurosurg 158:65–83 34718199 10.1016/j.wneu.2021.10.150

[30] Karakas C, Alam MC, Ferreira LD, Nair S, Kovalev D, Haneef Z (2025) Sociodemographic barriers in epilepsy surgery in the United States: a systematic review and meta-analysis. Epilepsy Behav 167:110391 40147221 10.1016/j.yebeh.2025.110391

[31] Amjad S, Tromburg C, Adesunkanmi M, Mawa J, Mahbub N, Campbell S et al (2024) Social determinants of health and pediatric emergency department outcomes: a systematic review and meta-analysis of observational studies. Ann Emerg Med 83(4):291–313 38069966 10.1016/j.annemergmed.2023.10.010

[32] Breeding T, Ngatuvai M, Rosander A, Maka P, Davis J, Knowlton LM et al (2023) Trends in disparities research on trauma and acute care surgery outcomes: a 10-year systematic review of articles published in the Journal of Trauma and Acute Care Surgery. J Trauma Acute Care Surg 95(5):806–815 37405809 10.1097/TA.0000000000004067

[33] Hannan EL, Van Ryn M, Burke J, Stone D, Kumar D, Arani D et al (1999) Access to coronary artery bypass surgery by race/ethnicity and gender among patients who are appropriate for surgery. Med Care 37(1):68–77 10413394 10.1097/00005650-199901000-00010

[34] Wood KL, Haider SF, Bui A, Leitman IM (2020) Access to common laparoscopic general surgical procedures: do racial disparities exist? Surg Endosc 34:1376–1386 31209603 10.1007/s00464-019-06912-w

[35] Butler PD, Familusi O, Serletti JM, Fox JP (2018) Influence of race, insurance status, and geographic access to plastic surgeons on immediate breast reconstruction rates. Am J Surg 215(6):987–994 29103529 10.1016/j.amjsurg.2017.09.037

[36] Johnson CT, Tran A, Preslar J, Bussey-Jones J, Schenker ML (2023) Racial disparities in the operative management of orthopedic trauma: a systematic review and meta-analysis. Am Surg 89(11):4521–4530 35981540 10.1177/00031348221121561

[37] Suneja N, Kong RM, Tiburzi HA, Shah NV, von Keudell AG, Harris MB et al (2022) Racial differences in orthopedic trauma surgery. Orthopedics 45(2):71–76 35021034 10.3928/01477447-20220105-02

[38] American Academy for Cererbal Palsy and Develomental Medicine (AACPDM). Hip Surveillance: Bottom Line "Evidence Informed" Recommendations for the Hip Surveillance in Individuals with Cerebral Palsy 2017 [Available from: https://www.aacpdm.org/publications/care-pathways/hip-surveillance-in-cerebral-palsy. Accessed 23 Aug 2025

